# Synergistic effects of TNF-alpha and melphalan in an isolated limb perfusion model of rat sarcoma: a histopathological, immunohistochemical and electron microscopical study.

**DOI:** 10.1038/bjc.1996.652

**Published:** 1996-12

**Authors:** P. T. Nooijen, E. R. Manusama, A. M. Eggermont, L. Schalkwijk, J. Stavast, R. L. Marquet, R. M. de Waal, D. J. Ruiter

**Affiliations:** Department of Pathology, University Hospital Nijmegen, The Netherlands.

## Abstract

**Images:**


British Journal of Cancer (1996) 74, 1908-1915
© 1996 Stockton Press All rights reserved 0007-0920/96 $12.00

### Synergistic effects of TNF-α and melphalan in an isolated limb perfusion model of rat sarcoma: a histopathological, immunohistochemical and electron microscopical study

PTGA Nooijen[1], ER Manusama[2], AMM Eggermont[2], L Schalkwijk[1], J Stavast[2], RL Marquet[2], RMW de Waal[1] and DJ Ruiter[1]

[1]*Department of Pathology, University Hospital Nijmegen, Nijmegen, The Netherlands;* [2]*Department of Surgery, University Hospital Rotterdam, Dr. Daniel den Hoed Cancer Centre, Rotterdam, The Netherlands.*

**Summary** Isolated limb perfusion (ILP) with tumour necrosis factor alpha (TNF-α) and melphalan has shown impressive results in patients with irresectable soft tissue sarcomas and stage III melanoma of the extremities. The mechanisms of the reported *in vivo* synergistic anti-tumour effects of TNF-α and melphalan are not precisely understood. We have developed an ILP model in the rat using a non-immunogenic sarcoma in which similar *in vivo* synergy is observed. The aim of this present study was to analyse the morphological substrate for this synergistic response of TNF-α in combination with melphalan to shed more light on the pathomechanisms involved. Histology of the tumours from saline- ($n=14$) and melphalan-treated ($n=11$) rats revealed apparently vital tumour cells in over 80% of the cross-sections. Interstitial oedema and coagulation necrosis were observed in the remaining part of the tumour. Haemorrhage was virtually absent. TNF-α ($n=22$) induced marked oedema, hyperaemia, vascular congestion, extravasation of erythrocytes and haemorrhagic necrosis (20–60% of the cross-sections). Oedema and haemorrhage suggested drastic alterations of permeability and integrity of the microvasculature. Using light and electron-microscopy, we observed that haemorrhage preceded generalised platelet aggregation. Therefore, we suggest that the observed platelet aggregation was the result of the microvascular damage rather than its initiator. Remarkably, these events hardly influenced tumour growth. However, perfusion with the combination of TNF-α and melphalan ($n=24$) showed more extensive haemorrhagic necrosis (80–90% of the cross-sections) and revealed a prolonged remission (mean 11 days) in comparison with the other groups of rats. Electron microscopical analysis revealed similar findings as described after TNF-α alone, although the effects were more prominent at all time points after perfusion. In conclusion, our findings suggest that the enhanced anti-tumour effect after the combination of TNF-α with melphalan results from potentiation of the TNF-α-induced vascular changes accompanied by increased vascular permeability and platelet aggregation. This may result in additive cytotoxicity or inhibition of growth of residual tumour cells.

**Keywords:** tumour necrosis factor alpha; melphalan; isolated limb perfusion; sarcoma; platelets

Isolated limb perfusion (ILP) with TNF-α and melphalan has shown impressive results in patients with irresectable soft tissue sarcomas and stage III melanoma of the extremities (Liénard *et al.*, 1992, 1994). ILP involves isolation of the diseased limb, its connection to a heart–lung machine and the administration of a triple drug regimen at 39–40°C (mild hyperthermia) based on the reported synergism of TNF-α with melphalan and interferon-γ (IFN-γ) (Eggermont *et al.*, 1993). The rationale of ILP is to improve the response rate by increasing the drug concentration while avoiding systemic toxicity. Morphological and immunohistochemical analysis of biopsies from patients after ILP suggested that the tumour microvasculature is a major target for TNF-α and melphalan. We and others have shown previously that events such as von Willebrand factor release, platelet aggregation and congestion concentrated on the tumour vasculature, leaving the normal tissues largely unaffected (Renard *et al.*, 1994, 1995). However, the mechanisms of *in vivo* synergistic anti-tumour effects of TNF-α and melphalan are still not understood precisely.

As the drug regimen in patients contains at least two experimental drugs, it does not allow us to reach definitive conclusions about the relative contribution of TNF-α and melphalan. Therefore, an experimental isolated perfusion model on sarcoma in the rat was devised. Rats transplanted with a non-immunogenic BN sarcoma in the hind leg were treated by isolated limb perfusion (Marquet *et al.*, 1983; Benckhuijsen *et al.*, 1982; Manusama *et al.*, 1994). Using this model, we recently showed *in vivo* synergism between TNF-α and melphalan in ILP with a tumour response resembling the clinical results (Manusama *et al.*, 1996). ILP in the rat with saline or 50 μg of TNF-α alone had no impact on tumour growth, and 40 μg of melphalan only temporarily inhibited tumour growth. In the group perfused with the combination of both TNF-α and melphalan, complete regression occurred in 75% of the rats. The aim of the present work was to study in this rat model the histopathological changes after ILP with TNF-α alone, melphalan alone and the combination of both drugs and so shed more light on the pathomechanisms involved. We hereby present evidence at the light and electron-microscopical level that the histopathological changes observed in the tumours after perfusion with TNF-α alone were augmented by addition of the chemotherapeutic agent.

Correspondence: P Nooijen, Department of Pathology, University Hospital Nijmegen, P.O. Box 9101, NL 6500 HB, Nijmegen, The Netherlands
Received 13 March 1996; revised 17 June 1996; accepted 2 July 1996

### Materials and methods

### *Animals*

Male rats of the inbred BN strain, weighing 250–300 g, were obtained from Harlan-CPB (Austerlitz, The Netherlands). They were fed a standard laboratory diet (Hope Farms, Woerden, The Netherlands) and kept under standard laboratory conditions. The specific protocol was approved by the committee on animal research of The Erasmus University, Rotterdam, The Netherlands.

*Tumour*

The spontaneous BN 175 sarcoma transplantable to BN rats was used (Marquet *et al.*, 1983). This BN 175 sarcoma is a rapidly growing and metastasising tumour. Immunogenicity of the tumour was determined by inoculating rats with tumour, excising the tumour, reinoculating the rats with the same tumour and measuring the percentage of rats that show tumour take. According to this method, described by Prehn and Main (1957), BN 175 sarcoma is non-immunogenic.

*Isolated limb perfusion (ILP)*

The tumour model and perfusion procedure were described previously by Manusama *et al.* (1994, 1996). Briefly, small fragments of BN 175 sarcoma (3–5 mm) were implanted in the right hind limb subcutaneously. Perfusion was performed in rats with established tumours with a mean diameter of 13.6±3.0 mm at 10.3±3.0 days after transplantation. Hypnorm (10 mg $ml^{-1}$ fluanisone, 0.315 mg $ml^{-1}$ fentanylcitrate: Janssen Pharmaceutica, Tilburg, The Netherlands) was given i.v. for anaesthesia. A warm water mattress was applied around the leg to maintain the temperature of the leg at 38–39°C. The temperature of the leg was measured during the perfusion by a naked bead type K probe fixed with its tip at the convexity of the tumour and connected to a digital thermometer (Mera Benelux, Berkel-Enschot, The Netherlands). The femoral artery and vein were approached by a parainguinal incision and cannulated in the distal direction with silastic tubing (artery: 0.30 mm inner diameter, 0.64 mm outer diameter, vein: 0.64 mm inner diameter, 1.19 mm outer diameter) (Dow, Corning, MI, USA). Collaterals of the femoral vessels were occluded during the perfusion by the application of a tourniquet in the groin. The tourniquet was fixed at the inguinal ligament. Isolation time commenced when the tourniquet was tightened. The circuit included an oxygenation chamber (5 ml syringe; Braun Melsungen, Germany) and a roller pump (type 505U; Watson Marlow, Falmouth, UK). The perfusion was started by circulating 5 ml of Haemaccel (Behring Pharma, Amsterdam, The Netherlands) resulting in a haemoglobin content of approximately 1 mmol $l^{-1}$ (mean 0.94±0.16). Melphalan (Wellcome, London, UK) and TNF-α (recombinant human TNF-α, (Boehringer Ingelheim, Germany) were added as boluses to the oxygenation resevoir. The treatment modalities are specified in Table I. The roller pump recirculated the perfusate at a flow rate of 2.4 ml $min^{-1}$, which was sufficient to maintain the partial pressure of oxygen, ($pO_2$) in the tumour (Manusama *et al.*, 1994). A washout was done at the end of the perfusion with 2.0 ml of oxygenated Haemaccel. The perfusion time was 30 min, including the washout. After the procedure, the femoral vessels of the perfused limb were ligated, which was allowed by restoration of the collateral circulation. In the BN rat, the presence of collateral circulation was demonstrated by a continuous venous return when the femoral artery was ligated. Furthermore, oxygen pressure in the tumour after ligation of the femoral vessels was equivalent to that before ligation as described in a previous study (Manusama *et al.*, 1994).

**Table I** Specification of the treatment modalities

| *Treatment (modalities)* | *Sacrifice (hours after perfusion)* | *Number of rats* |
|---|---|---|
| Controls (without perfusion) | 0 | 5 |
| Saline | 2 | 4 |
| | 4 | 5 |
| | 12 | 1 |
| | 24 | 3 |
| | 72 | 1 |
| Melphalan (40 μg) | 2 | 3 |
| | 4 | 4 |
| | 12 | 1 |
| | 24 | 3 |
| TNF-α (50 μg) | 2 | 5 |
| | 4 | 6 |
| | 12 | 3 |
| | 24 | 6 |
| | 72 | 2 |
| Melphalan and TNF-α (40 μg and 50 μg respectively) | 2 | 4 |
| | 4 | 6 |
| | 12 | 3 |
| | 24 | 8 |
| | 72 | 3 |

**Figure 1** Degree of tumour necrosis after various treatment regimens, expressed as percentage of the cross-section.

### *Histological procedure*

The tumours were excised with a rim of skin whereas the muscle layer formed the deep resection margin. After removal, the tumours were carefully cut in two almost equal parts in dorsoventral direction. In 42 out of 76 rats one half of the specimen was directly frozen in liquid nitrogen and stored at −80°C until further processing and the other half was divided into a peripheral part (containing the margin tumour/pre-existing tissues) and a central part (containing

**Figure 2** Paraffin sections of ILP-treated rat BN sarcoma, haematoxylin–eosin (HE) stained. (**a**) Overview of tumour and skin, 24 h after ILP with saline (magnification ×40). (**b**) Tumour, 24 h after ILP with saline (magnification ×200, detail from **a**). A blood vessel is marked by an arrow and a mitotic figure by an arrowhead. (**c**) Overview of tumour and skin, 24 h after ILP with melphalan (magnification ×40). (**d**) Tumour, 24 h after ILP with melphalan (magnification ×200, detail from **c**). (**e**) Overview of tumour and skin, 24 h after ILP with TNF-α (magnification ×40). (**f**) Tumour, 24 h after ILP with TNF-α (magnification ×100, detail from 1e). Haemorrhage is marked by an arrow. (*g*) Overview of tumour and skin, 24 h after ILP with the combination of TNF-α and melphalan (magnification ×40). (**h**) Tumour, 24 h after ILP with the combination of TNF-α and melphalan (magnification ×100, detail from **g**). A blood vessel with a thrombus is marked by an arrow. s, Skin; t, tumour; n, necrosis; h, haemorrhage.

tumour from the centre of the lesion) and prepared for ultrastructural analysis. In 34 rats, both parts of the specimen were formalin-fixed and embedded in paraffin. Cyrostat and paraffin sections (4 $\mu$m) were haematoxylin–eosin stained. Tumour necrosis was assessed by conventional histological criteria; the percentage of non-viable tumour was estimated in a representative histological section that included both the central and the peripheral borders of the tumour bed and expressed as percentage of the cross-section and scored in the following categories: 0–20%, 21–50%, 51–80% and 81–100%. The extent and character of the inflammatory infiltrate was evaluated. The slides were read by two different observers. In case of disagreement, consensus was reached during joined re-examination.

*Immunohistochemistry*

Platelet aggregation was visualised by immunohistochemistry on representative cryostat sections that included both the central and the peripheral border of the tumour bed, 4, 12 and 24 h after perfusion with saline ($n=4$), melphalan ($n=4$), TNF-$\alpha$ ($n=6$) and TNF-$\alpha$ in combination with melphalan ($n=6$). Cryostat sections (4 $\mu$m) were stained using a two-step immunoperoxidase procedure, as described previously (Nooijen *et al.*, 1996), using MAb PL1-1/Er 21 (Baghus *et al.*, 1989) directed against rat platelets, kindly provided by Dr E De Heer (University Hospital Leiden, Leiden, The Netherlands), and using PAb RaHu FVIII, recognising von Willebrand factor (VWF) from the Central Laboratory of the Netherlands Red Cross Blood Transfusion Service (CLB). As secondary antibodies, peroxidase-conjugated swine anti-rabbit Ig and rabbit anti-mouse Ig were used (Dako, 1:100), preabsorbed with 5% normal rat serum. The peroxidase label was visualised by incubation with 3-amino-9-ethylcarbazole as a substrate. The semiquantitative grading used was as follows: no change (o), sporadic event ($\pm$), focal event (+), generalised event (+ +).

*Electron microscopy*

Small tissue fragments from the central and the peripheral part of the tumour were immediately fixed for 24 h in 2.5% glutaraldehyde with 0.1 mol sodium cacodylate. The material was post-fixed in 1% osmium sodium cacodylate buffer for 1 h at room temperature (RT), dehydrated and embedded in epon 812. One micron sections were cut and stained with toluidine blue for light microscopy. Ultrathin sections were cut with a diamond knife (Drukker, Cuijk, The Netherlands) on an ultramicrotome (Reichert Jung, Vienna, Austria). The ultrathin sections were contrasted for 15 min with uranyl 3%, followed by a 3 min treatment with lead citrate, and examined and photographed with a JEOL 1200 EX/II electron microscope (Tokyo, Japan) at 60 kV. The electron microscopical analysis focused on the microvascular changes.

### Results

*Histology*

The semiquantitative assessment of tumour necrosis in a representative histological section after the different treatment modalities at various time points following the perfusion is shown in Figure 1.

*Controls* Slices of tumours from saline-treated and untreated control rats showed the presence of solid and grey tumour mass between the epidermis and the muscle of the right hind limbs. Histology revealed individual cell necrosis and scattered areas of confluent necrosis, both of the coagulative type. Over 80% of the tumour consisted of apparently vital tumour tissue with several mitotic figures and showing a high vascularity (Figure 2a and b). Apoptotic bodies were seen incidentally. Hyperaemia and oedema were observed locally. Scattered mononuclear inflammatory cells were noted in the tumour and at the interface of tumour with dermal and subcutaneous tissues (not shown). Margination of polymorphonuclear cells was apparent in dilated vessels next to the tumour (not shown).

*Melphalan* Slices of tumours treated with melphalan generally appeared solid and grey. Microscopical examination 24 h after melphalan showed a highly cellular tumour mass (Figure 2c and d). Over 80% of the cut surface of the tumour sections consisted of apparently vital tumour tissue. Interstitial oedema, scattered areas of necrosis and individual cell necrosis were observed in the remaining part of the tumour. Necrosis was of the coagulative type. Tumour cells with fragmented nuclei were encountered, compatible with apoptosis. Haemorrhage was virtually absent. The tumour

**Figure 3** Semiquantitative assessment of platelet aggregation after various treatment regimens, in a representative cryostat section. O, No change; $\pm$, sporadic event; +, focal event; + +, generalised event.

mass was hardly infiltrated by polymorphonuclear cells, but scattered mononuclear inflammatory cells were observed in the centre and at the margin.

*TNF-α* Slices of tumours from animals treated with TNF-α generally appeared red and soft in comparison with the solid grey tumours in the untreated and saline-treated controls. Histology of the material harvested 2 h after ILP with TNF-α revealed vascular congestion, marked interstitial oedema and focal extravasation of erythrocytes (not shown). These vascular effects were most obvious in the tumour margins and in the adjacent connective tissue. Four hours after ILP, TNF-α induced a red discoloration with diffuse haemorrhage and marked vascular congestion (defined as dilated vessels compacted with erythrocytes as a sign of hyperaemia and/or haemostasis) compared with the controls, both in the central parts of the tumour and at the interface of the tumour with dermal tissues (not shown). Twenty-four hours after TNF-α treatment, haemorrhage and tumour cell necrosis could be observed centrally and constituted 20–60% of the tumour (Figure 2e and f). Vascular congestion and thrombi were often seen. Histologically vital-appearing tumour cells were situated at the margins next to normal skin. In and around areas with haemorrhage, scattered mononuclear inflammatory cells could be observed. Infiltration of the tumour by polymorphonuclear cells was seen in four rats (two rats 4 h after ILP and two rats 24 h after ILP) (not shown).

*Combination of TNF-α and melphalan* Slices of tumours from animals treated with the combination of drugs generally appeared red and soft. Microscopic examination 2 h after ILP revealed marked vascular congestion and interstitial oedema along with extravasation of erythrocytes (not shown). Scattered mononuclear inflammatory cells were present in the tumour and at the periphery. Margination of polymorphonuclear cells was observed in a few cases in some dermal vessels next to the tumour. At 4 h after perfusion, vascular congestion and haemorrhage were generally seen (not shown). Twelve hours after ILP these effects were intensified with increased disintegration of tumour cells, showing apparent nuclear pyknosis or fragmentation. These effects were more prominent centrally in the tumour. By 24 h, approximately 80–90% of the tumour had undergone extensive necrosis. Cell debris, oedema, haemorrhage, thrombi and mononuclear inflammatory cells were observed (Figure 2g and h). In three rats (out of 24), a moderate infiltration of the tumour by polymorphonuclear cells was observed (not shown). In addition, the epidermis overlying the area of central necrosis was necrotic (Figure 2g). A rim of viable tumour cells persisted at the margins next to the dermis (Figure 2g and h) and seemed to be responsible for the outgrowth of the tumour over the subsequent days.

### *Immunohistochemistry*

*PL1-1 (platelets)* The semiquantitative assessment of platelet aggregation in a representative histological section after various treatment modalities at various time points following perfusion is shown in Figure 3. Sections of the saline-treated rats 4, 12 and 24 h after perfusion showed sporadic (±) intravascular PL1-1 staining in the tumour (Figure 4a). PL1-1 staining 4, 12 and 24 h after melphalan treatment varied from sporadic (±) to focal (+) (Figure 4b). PL1-1 staining in the vessels outside the tumour was sporadically observed. TNF-α-treated rats showed intravascular PL1-1 staining focally (+) in the tumour 4 h after treatment (not shown). Generalised (+ +) PL1-1 staining was observed 12 and 24 h after TNF-α-perfusion (Figure 4c). PL1-1 staining was also observed in vessels adjacent to the tumour. Perfusion treatment with TNF-α in combination with melphalan showed intravascular PL1-1 staining focally (+) in the tumour in sections 4 h after perfusion (not shown). In all sections 12 and 24 h after perfusion with TNF-

**Figure 4** Immunohistochemical staining with the platelet marker PL1-1 on frozen sections of rat BN sarcoma. (**a**) Overview of tumour, 12 h after ILP with saline (magnification ×50) showing sporadic (±) PL1-1 staining. (**b**) Overview of tumour, 12 h after ILP with melphalan (magnification ×50) showing focal (+) PL1-1 staining. (**c**) Overview of tumour, 12 h after ILP with TNF-α (magnification ×50) showing generalised (+ +) PL1-1 staining. (**d**) Overview of tumour, 12 h after ILP with TNF-α and melphalan (magnification ×50) showing marked generalised (+ +) PL1-1 staining. c, Tumour centre; p, periphery of the tumour.

α in combination with melphalan, marked generalised (+ +) PL1-1 staining was observed (Figure 4d), and also in vessels adjacent to the tumour (Figure 4e).

*rHu FVIII (von Willebrand factor)* We found that this antibody was not suitable for the analysis as possible leakage of von Willebrand factor by the endothelium after perfusion treatment in our animal model, as the intensity of the endothelial staining was low in combination with a diffuse background staining.

### *Electronmicroscopy*

The tumours contained numerous small vessels. Intratumoral vessels at the periphery of the control tumours and after saline perfusion revealed a continuous endothelial cell lining, often surrounded by pericytes. Smooth muscle cells were only occasionally found. Centrally in the tumour, few degenerated endothelial cells were observed in areas with oedema and haemorrhage, suggestive of vascular leakage. These findings were not accompanied by a marked inflammatory cell infiltrate. Platelets were found adherent to the vessel wall. The ultrastructural findings after ILP with melphalan (40 μg) resembled those described after saline perfusion.

After perfusion with TNF-α (50 μg), marked intercellular oedema was seen. Increased haemorrhage, both centrally and at the periphery (2, 4 and 12 h after ILP), suggested vascular leakage (Figure 5b). Intratumoral and peritumoral vessels showed marked erythrostasis (Figure 5a and 5f). At the periphery of the tumours, the vessels loaded with erythrocytes revealed signs of endothelial cell degeneration, i.e. electron-lucent cytoplasm and swollen mitochondria (Figure 5a and c). Extensive aggregation of platelets was observed both in the intratumoral vessels and just outside the tumour, but merely mural and not occlusive (Figure 5e). Increased haemorrhage and intravascular platelet aggregation were observed 24 h after perfusion with TNF-α in the tumour, with disintegration of the endothelial cells (Figure 5d), only in the tumour centre, with extensive oedema and tumour cell necrosis. These phenomena were not accompanied by a marked inflammatory cell infiltrate.

Ultrastructural analysis of sections after perfusions with TNF-α and melphalan revealed similar findings as described after TNF-α alone, although the findings were more prominent at all time points after perfusion. Because of this similarity, only the ultrastructural changes after TNF-α are shown.

**Figure 5** Ultrastructural microvascular changes after ILP with TNF-α. (**a**) Blood vessel at the periphery of the tumour, 12 h after ILP, loaded with erythrocytes and lined by degenerated endothelial cells (magnification ×4000). (**b**) Erythrocyte extravasation in the periphery of the tumour, 4 h after ILP (magnification ×5000). (**c**) Detail of the endothelial cell in Figure 3a, showing signs of degeneration. A swollen mitochondrion is marked by an arrow (magnification ×6000). (**d**) Blood vessel in the tumour centre, 12 h after ILP showing disturbed integrity of the endothelial cell (marked by an arrow) (magnification ×2500). Inset, high power image of part of the cytoplasm of the endothelial cell showing merely intact mitochondria. (**e**) Platelet aggregation in a blood vessel in the periphery of the tumour, 12 h after ILP (magnification ×6000). (**f**) Blood vessel in the dermis adjacent to the tumour, 12 h after ILP, loaded with erythrocytes. E, erythrocytes; EC, endothelial cell; BM, basement membrane; P, platelet.

### Discussion

Tumour necrosis factor-α (TNF-α) in combination with interferon-γ (IFN-γ) and melphalan in an isolated limb perfusion setting resulted in a high remission rate in patients with irresectable soft tissue sarcomas (Liénard *et al.*, 1992; Eggermont *et al.*, 1992). An overall response rate of 88% with a limb salvage of 87% was reported (Eggermont *et al.*, 1993). However, perfusion with TNF-α alone proved to be ineffective (Posner *et al.*, 1994). The mechanism of tumour regression by the combination of TNF-α and the chemotherapeutic agent melphalan, *in vivo*, is not precisely understood but was proposed to follow a dual targeting pathway (Lejeune, 1995; Renard *et al.*, 1995). The first target is represented by the tumour microvasculature. TNF-α was assumed to induce endothelial cell damage, leading to von Willebrand factor release (VWF). Released VWF may play a role in the adhesion between platelet and the damaged endothelium or the denuded vessel wall. As a consequence, the blood flow is impaired, leading to congestion and oedema. The second target is represented by the tumour cells themselves, which are increasingly subjected to the cytotoxic effects of melphalan in a hypoxic environment.

In order to study the impact of the individual drugs on tumour regression we used an experimental model of sarcoma in the rat. We previously showed an *in vivo* synergism between two relatively ineffective doses of TNF-α (50 μg) and melphalan (40 μg) in this rat model, with a tumour response resembling the clinical response (Manusama *et al.*, 1996). The present study was set up to analyse the morphological substrate for this synergistic response employing light microscopic, immunohistochemical and electron microscopic methods. Histopathological analysis of the transplanted non-immunogenic BN sarcoma after ILP with TNF-α alone showed similar findings as reported by Asher *et al.* (1987) in a non-immunogenic MCA-102 murine sarcoma after TNF-α i.v. injection and closely resembled the effects described for endotoxin-induced histopathological features (van de Wiel *et al.*, 1989; Kuper *et al.*, 1985, 1986). TNF-α induced oedema, hyperaemia, vascular congestion, extravasation of erythrocytes and haemorrhagic necrosis (20–60%) (Regenass *et al.*, 1987; MacPherson and North, 1986; Sato *et al.*, 1986). Remarkably, these events hardly influenced tumour diameter and tumour growth in our rat ILP model. Increased doses of only TNF-α did not lead to an increased anti-tumour effect; even with doses of 100 μg in all seven rats, progressive disease was observed (unpublished results). Furthermore, when using TNF-α intravenously in toxic doses, no effect on tumour growth was noted in the same model (unpublished results). Therefore, the combination with melphalan is necessary to obtain an effective anti-tumour response. On the other hand, at higher doses of melphalan alone, regression occurred with a gradual disappearance of the tumour without haemorrhagic necrosis (Manusama *et al.*, 1996). Combination with TNF-α lowered the effective dose of melphalan and introduced a vascular component into the mechanism of action, characterised by extensive haemorrhagic necrosis.

Oedema and haemorrhage suggested dramatic alterations of permeability and integrity of microvascular endothelial cells. The cause of the vascular stasis and congestion is not clear, but it may be related to a direct cytotoxic effect of TNF-α on endothelial cells (Sato *et al.*, 1986). A direct toxic effect and activation of procoagulant activity in tumour endothelial cells with subsequent thrombus formation might be responsible for haemorrhagic necrosis (Gerlach *et al.*, 1989; Watanabe *et al.*, 1988; Nawroth *et al.*, 1988; Shimomura *et al.*, 1988). Using light and electron microscopy, we observed, however, that haemorrhage preceded generalised platelet aggregation without fusion of platelets with endothelial cells (Grau *et al.*, 1993). Therefore, we suggest that the observed platelet aggregation was the result of the endothelial damage rather than the initiator. These findings are consistent with those observed in sequential biopsies of human lesions after ILP (Renard *et al.*, 1995). In most cases, the centre of the tumour seemed to be more vulnerable to the treatment than the periphery. Aberrant branching and twisting of the vasculature and abnormal high pressure in the interstitial matrix of the tumour centre, with pre-existing increased vascular permeability, may have led to an uneven distribution of the drugs and uneven distribution of the vascular changes observed (Jain, 1987*a*,*b*). The vascular changes in the tumour margins, accompanied with stasis, may have contributed to a decreased blood flow out of the tumour with development of central haemorrhagic necrosis.

The anti-tumour activity and necrosis induced by the combination of intratumorally injected TNF-α and IFN-γ systemically were studied by de Kossodo *et al.* (1995) in a breast cancer xenograft model. Recent study in an experimental ILP model revealed almost similar findings using the combination of TNF-α and melphalan. Similar potentiation of the anti-tumour activity were observed, associated with vascular congestion and accumulation of platelets in areas of vascular damage. Whether polymorphonuclear leucocytes contributed to the vascular damage in our rat ILP model is not clear. A polymorphonuclear cell infiltrate in the tumour after perfusion with TNF-α and with the combination generally was not observed, whereas haemorrhage and necrosis were a consistent finding. The exact role of polymorphonuclear leucocytes in the anti-tumour effect needs to be analysed in future studies using perfusions of granulocytopenic rats. Although it is difficult to derive the dynamics of vascular damage and tumour regression from static images, our observations support the interpretation of Regenass *et al.* (1987) that the TNF-α-induced increase in permeability of the endothelial cells leads to increased blood viscosity. In this view, the decreasing tumour blood flow is further impaired by intravascular platelet aggregation, resulting in a sustained haemostasis and finally in haemorrhagic infarction. The increased vascular permeability together with a reduced blood flow out of the tumour may have led to increased intratumoral concentrations of melphalan or prolongation of its effect. Our findings indicate that it is important to measure the effects on vascular permeability. Experiments to determine vascular permeability changes and melphalan concentrations in the tumours are in progress.

Perfusion with the combination of TNF-α and melphalan increased both the occurrence and extent of the haemorrhagic necrosis. Necrosis was not confined to the tumour cells but also involved microvascular cells, both in the tumour and in the adjacent dermis. The potentiation of the TNF-α-induced vascular effects by melphalan might be explained by additive cytotoxicity direct to the vascular endothelium (Regenass *et al.*, 1987; Kachel and Martin, 1994; Alexander *et al.*, 1987) and/or increased endothelial cell reactivity to platelets (Bertomen *et al.*, 1990). Our (histo)pathological findings support the proposition by Lejeune (1995) that the combination of TNF-α and melphalan works through a dual targeting system. In this view, the first target is represented by the tumour vasculature and the second one by the tumour cells themselves. However, the proposed granulocyte-mediated endothelial damage does not seem to be a *conditio sine qua non* and therefore should be studied further. In conclusion, our findings suggest that the enhanced anti-tumour effect after the combination of TNF-α with melphalan results from potentiation of the TNF-α-induced vascular changes accompanied by increased permeability. This may result in additive cytotoxicity or inhibition of growth of residual tumour cells.

**Acknowledgements**
This study was supported by a grant from the Dutch Cancer Society (NKB 93-659). We thank FJR Rietveld for his expert technical assistance with the electron microscopical analysis and NMC Durante for his expert technical assistance with the perfusion procedure. We thank Boehringer Ingelheim, Germany, for providing TNF-α.

### References

ALEXANDER RB, NELSON WG AND GOFFEY DS. (1987). Synergistic enhancement by tumour necrosis factor of in vitro cytotoxicity from chemotherapeutic drugs targeted at DNA topoisomerase II. *Cancer Res.*, **47**, 2403–2406.

ASHER A, MULÉ JJ, REICHERT CM, SHILONI E AND ROSENBERG SA. (1987). Studies on the anti-tumour efficacy of systemically administered recombinant tumour necrosis factor against several murine tumours in vivo. *J. Immunol.*, **138**, 963–974.

BAGHUS WM, JEUNINK MF, ROZING J AND ELEMA JW. (1989). A monoclonal antibody against rat platelets. I. Tissue distribution in vitro and in vivo. *Clin. Exp. Immunol.*, **75** 317–323.

BENCKHUIJSEN C, VAN DIJK WS AND VAN HET HOFF SC. (1982). High flow isolation perfusion of the rat hind limb in vivo. *J. Surg. Oncol.*, **21**, 249–257.

BERTOMEN MC, GALLO S, LAURI D, LEVINE, MN, ORR FW AND BUCHANAN MR. (1990). Chemotherapy enhances endothelial cell reactivity to platelets. *Clin. Expl. Metastasis*, **8**, 511–518.

EGGERMONT AMM, LIÉNARD D, SCHRAFFORDT KOOPS H, ROSEN-KAIMER F AND LEUJEUNE FJ. (1993). Treatment of irresectable soft-tissue sarcomas of the limbs with high dose TNF-α in combination with interferon-γ and melphalan. In *Tumour Necrosis Factor: Molecular and Cellular Biology and Clinical Relevance*, Fiers W and Buurman W. (eds) pp. 239–243. Karger: Basle.

GERLACH H., LIBERMAN H, BACH R, GODMAN G, BRETT J AND STERN D. (1989). Enhanced responsiveness of endothelium in the growing/motile state to tumour necrosis factor/cachectin. *J. Exp. Med.*, **170**, 913–931.

GRAU GE, TACCHINI-COTTIER F, VESIN C, MILON G, LOU JN, PIGUET PF AND JUILLARD P. (1993). TNF-induced microvascular pathology: active role for platelets and importance of the LFA-1/ICAM-1 interaction. *Eur. Cytokine Netw.*, **4**, 415-419.

JAIN RK. (1987*a*). Transport of molecules across tumor vasculature. *Cancer Metastasis Rev.*, **6**, 559-593.

JAIN RK. (1987*b*). Transport of molecules in the tumor interstitium: A review. *Cancer Res.*, **47**, 3039-3051.

KACHEL DL AND MARTIN WJ. (1994). Cyclophosphamide-induced lung toxicity: mechanisms of endothelial cell injury. *J. Pharmacol. Exp. Ther.*, **268**, 42-46.

DE KOSSODO S, MOORE R, GSCHMEISSNER S, EAST N, UPTON C AND BALKWILL FR. (1995). Changes in endogenous cytokines, adhesion molecules and platelets during cytokine-induced tumour necrosis. *Br. J. Cancer*, **72**, 1165-1172.

KUPER F, BLOKSMA N, BRUIJNTJES JP, HOFHUIS FMA AND WOLTERINK G. (1985). Effects of endotoxin-treatment on inflammatory cell infiltrates in murine meth-A sarcoma. *J. Pathol.*, **147**, 41-48.

KUPER F, BLOKSMA N AND HENDRIKSEN EGJ. (1986). *Antitumour Effects of Endotoxin Against Solid Murine Meth A Tumours of Different Ages. II. Ultrastructure of Vascular and perivascular events.* PhD thesis, State University Utrecht, The Netherlands.

LEJEUNE FJ. (1995). High dose recombinant tumour necrosis factor (rTNF-α) administered by isolation perfusion for advanced tumours of the limbs: a model for biochemotherapy of cancer. *Eur. J. Cancer*, **31a** 1009-1016.

LIÉNARD D, DELMOTTE JJ, RENARD N, EWALENKO P AND LEJEUNE FJ. (1992). High doses of rTNF-α in combination with IFN-gamma and melphalan in isolation perfusion of the limbs for melanoma and sarcoma.*J. Clin. Oncol.*, **10**, 52-60.

LIÉNARD D, EGGERMONT AMM, SCHRAFFORDT KOOPS H, KROON BB, ROSENKAIMER F, AUTIER P AND LEJEUNE FJ. (1994). Isolated limb perfusion of the limb with high-dose tumour necrosis factor-alpha (TNF–alpha), interferon-gamma (INF-gamma) and melphalan for melanoma stage III. Results of a multi-center pilot study. *Melanoma Res.*, **4** (suppl. 1), 21-26.

MACPHERSON GG AND NORTH RJ. (1986). Endotoxin mediated necrosis and regression of established tumours in the mouse. A correlative study of quantitative changes in blood flow and ultrastructural morphology. *Cancer Immunol. Immunother.*, **21**, 209-216.

MANUSAMA ER, DURANTE NMC, MARQUET RL AND EGGERMONT AMM. (1994). Ischemia promotes the antitumour effect of tumour necrosis factor alpha (TNF-α) in isolated limb perfusion in the rat. *Reg. Cancer Treat.*, **7**, 155-159.

MANUSAMA ER, NOOIJEN PTGA, STAVAST J, DURANTE NMC, MARQUET RL AND EGGERMONT AMM. (1996). Synergistic antitumour effect of recombinant human tumour necrosis factor alpha (TNF-α) with melpahalan in isolated limb perfusion in the rat. *Br. J. Surgery*, **83**, 551-555.

MARQUET RL, SCHELLEKENSH, WESTBROEK DL AND JEEKEL J. (1983). Effect of treatment with interferon and cyclophosphamide on the growth of a spontaneous liposarcoma in rats. *Int. J. Cancer*, **31**, 223-226.

NAWROTH PD, HANDLEY H, BACH R, GODMAN G, BRETT J AND STERN D. (1989). Enhanced responsiveness of endothelium in the growing/motile state to tumour necrosis factor/cachectin. *J. Exp. Med.*, **170**, 913-931.

NOOIJEN PTGA, EGGERMONT AMM, VERBEEK MM, SCHALKWIJK L, BUURMAN WA, DE WAAL RMW AND RUITER DJ. (1996). Transient induction of E-selectin expression following TNF-α based isolated limb perfusion in melanoma and sarcoma patients is not tumour-specific. *J. Immunother.*, **19**(I), 33-44.

POSNER M, LIÉNARD D, LEJEUNE FJ, ROSENFELDER D AND KIRKWOOD J. (1994). Hyperthermic isolated limb perfusion (HILP) with tumour necrosis factor (TNF) alone for metastatic in transit melanoma. *Proc. Am. Soc. Clin. Oncol.*, **13**, 369.

PREHN RT AND MAIN JM. (1957). Immunity to methylcholanthrene-induced sarcomas. *J. Natl Cancer Inst.*, **18**, 769-778.

REGENASS U, MÜLLER M, CURSCHELLAS E AND MATTER A. (1987). Anti-tumour effects of tumour necrosis factor in combination with chemotherapeutic agents. *Int. J. Cancer*, **39**, 266-273.

RENARD N, LIÉNARD D, LESPAGNARD L, EGGERMONT AMM, HEIMANN R AND LEJEUNE FJ. (1994). Early endothelium activation and polymorphnuclear cell invasion precedes specific necrosis of human melamona and sarcoma treated by intravascular high-dose tumour necrosis factor alpha (rTNFα). *Int. J. Cancer*, **57**, 656-663.

RENARD N, NOOIJEN PTGA, SCHALKWIJK L, DE WAAL RMW, EGGERMONT AMM, LIÉNARD D, KROON BBR, LEJEUNE FJ AND RUITER DJ. (1995). VWF release and platelet aggregation in human melanoma after perfusion with TNF-α. *J. Pathol.*, **176**, 279-287.

SATO N, GOTO T, HARANAKA K, SATOMI N, NARIUCHI H, MANOHIRANO Y AND SASWASKI Y. (1986). Actions of tumour necrosis factor on cultured vascular endothelial cells: morphologic modulation, growth inhibition, and cytotoxicity. *J. Natl Cancer Inst.*, **76**, 1113-1121.

SHIMOMURA K, MANDA T, MUKUMOTO S, KOBAYASHI K, NAHANO K AND MORI J. (1988). Recombinant human tumour necrosis factor-α: thrombus formation is a cause of anti-tumour activity. *Int. J. Cancer*, **41**, 243-247.

WATANABE N, NIITSU Y AND UMENO H. (1988). Toxic effects of tumor necrosis factor on tumor vasculature in mice. *Cancer Res.*, **48**, 2197-2183.

WIEL VAN DE PA, BLOKSMA N, KUPER CF, HOFHUIS FMA AND WILLERS JMN. (1989). Macroscopic and microscopic early effects of tumour necrosis factor on murine Meth A Sarcoma, and relation to curative activity. *J. Pathol.*, **157**, 65-73.


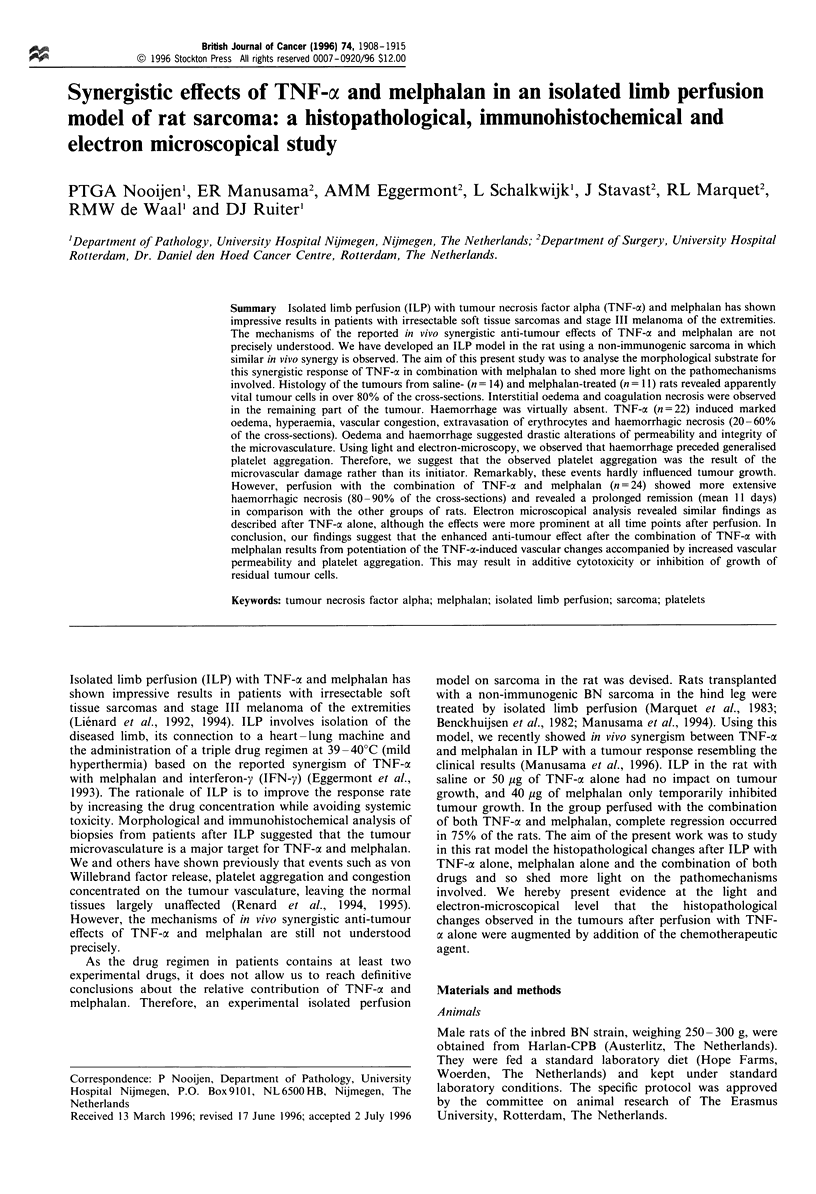

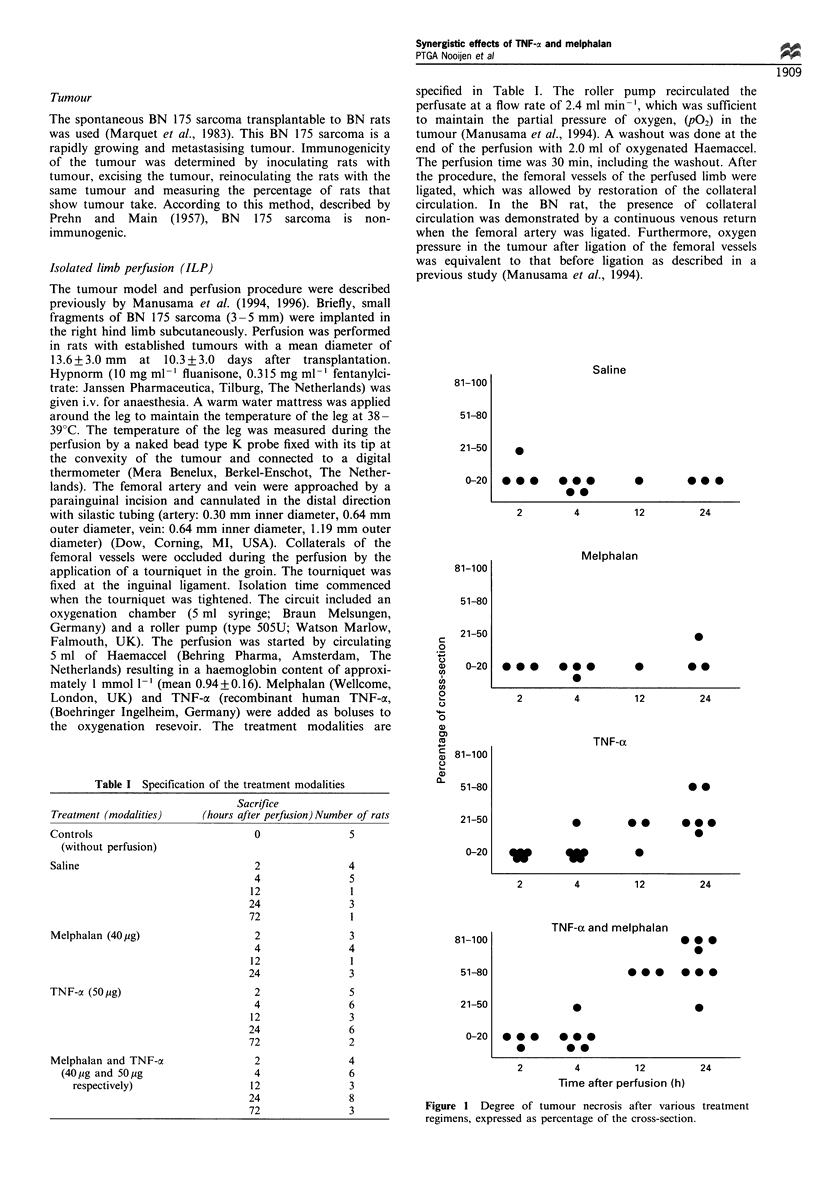

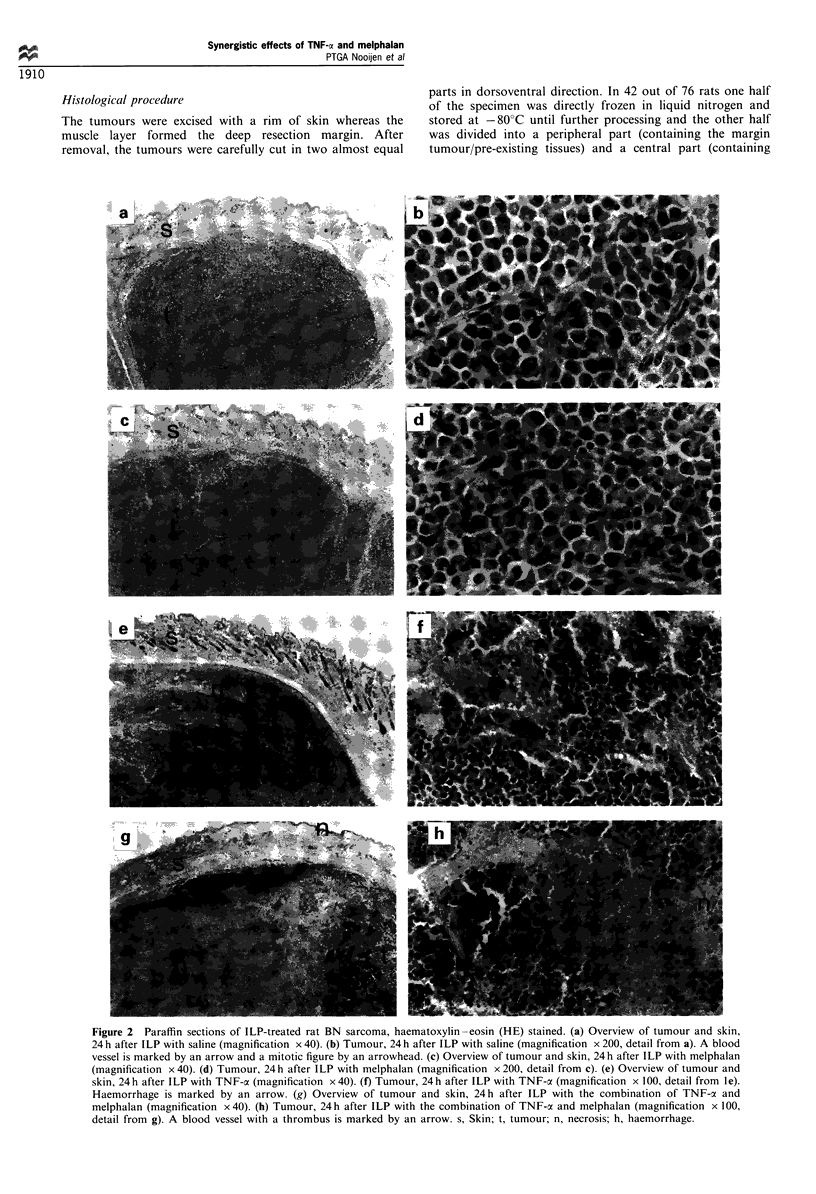

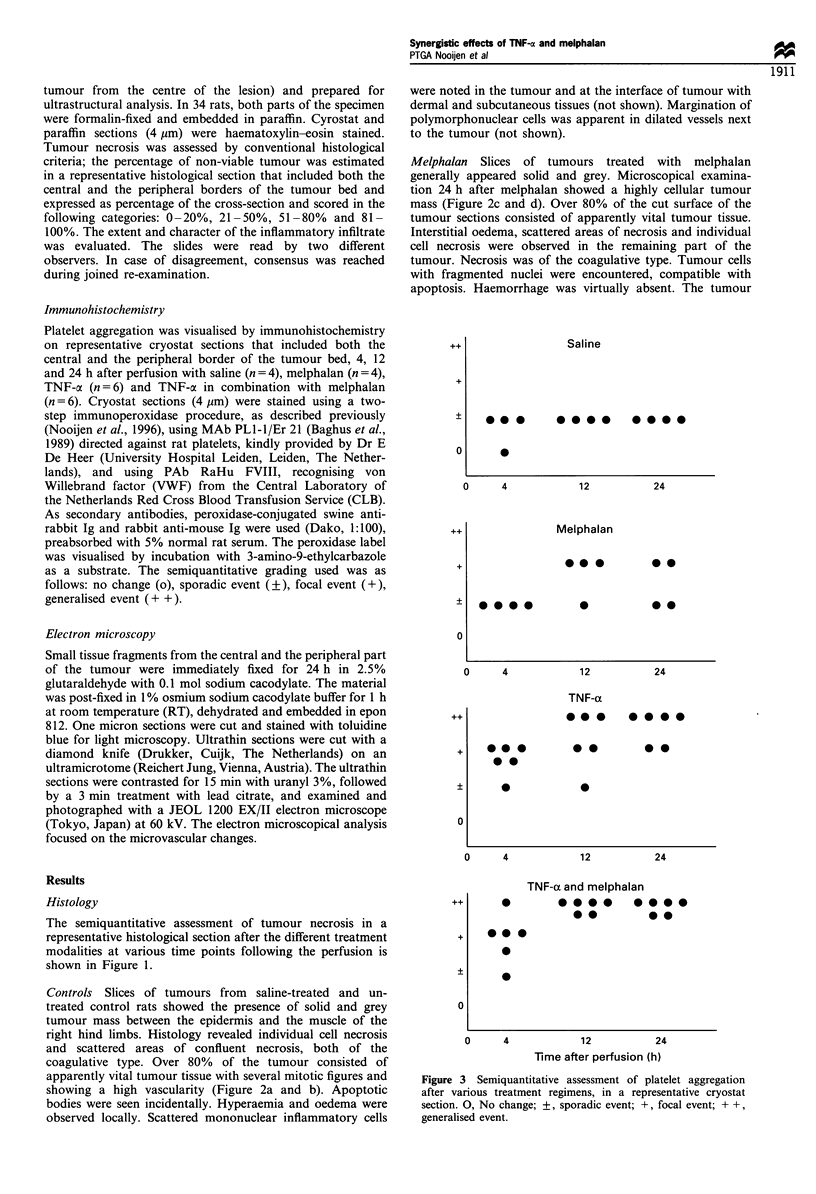

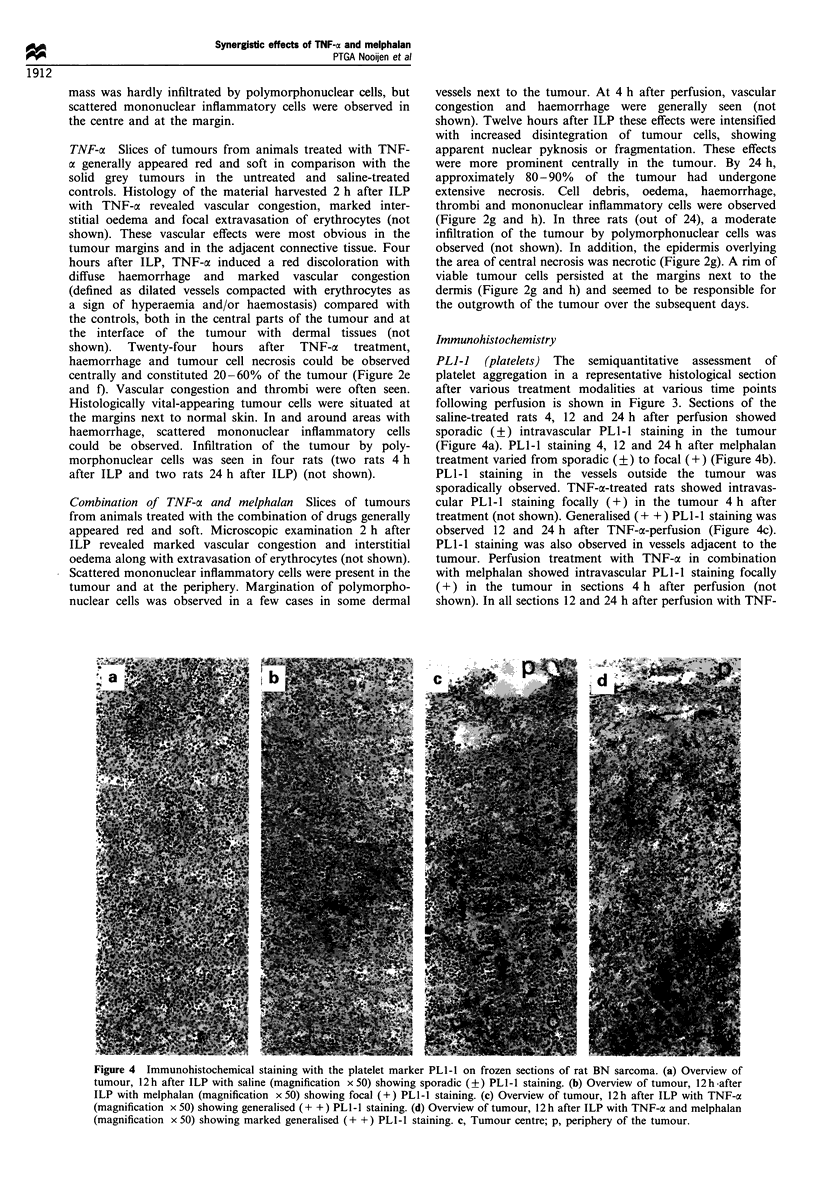

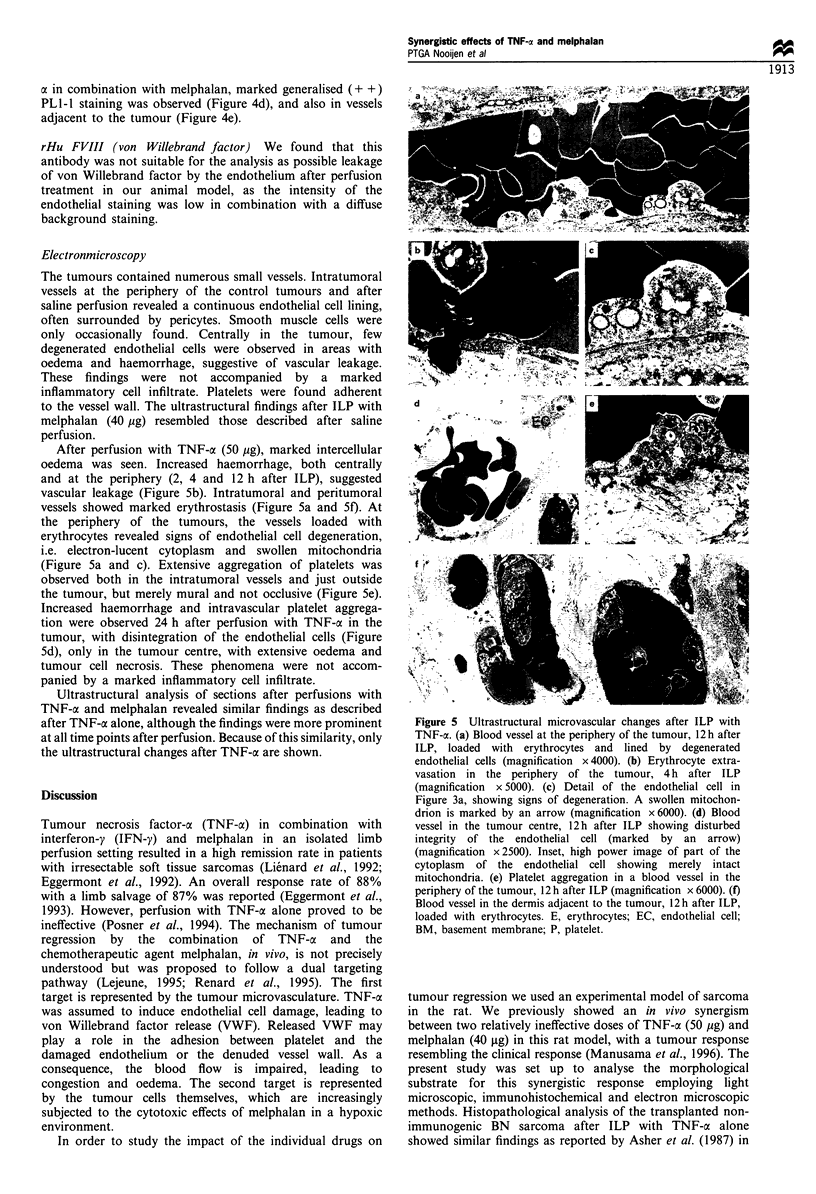

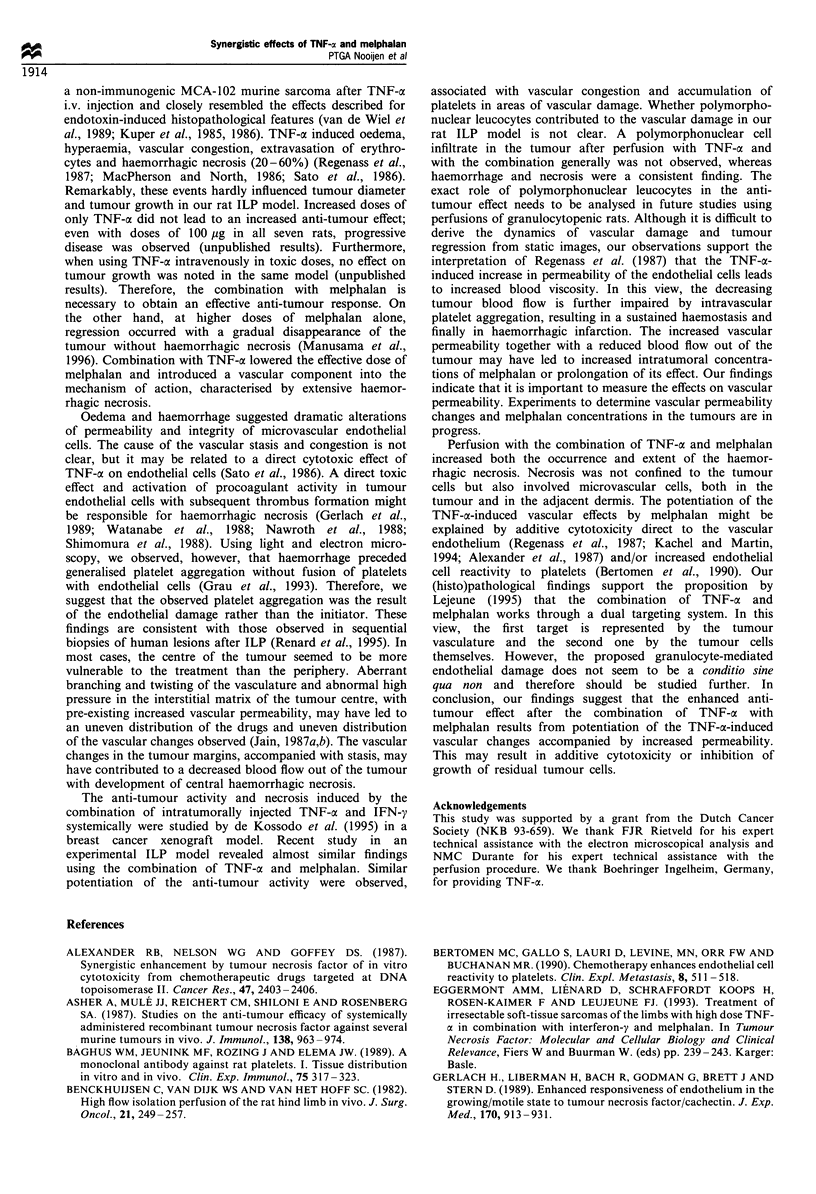

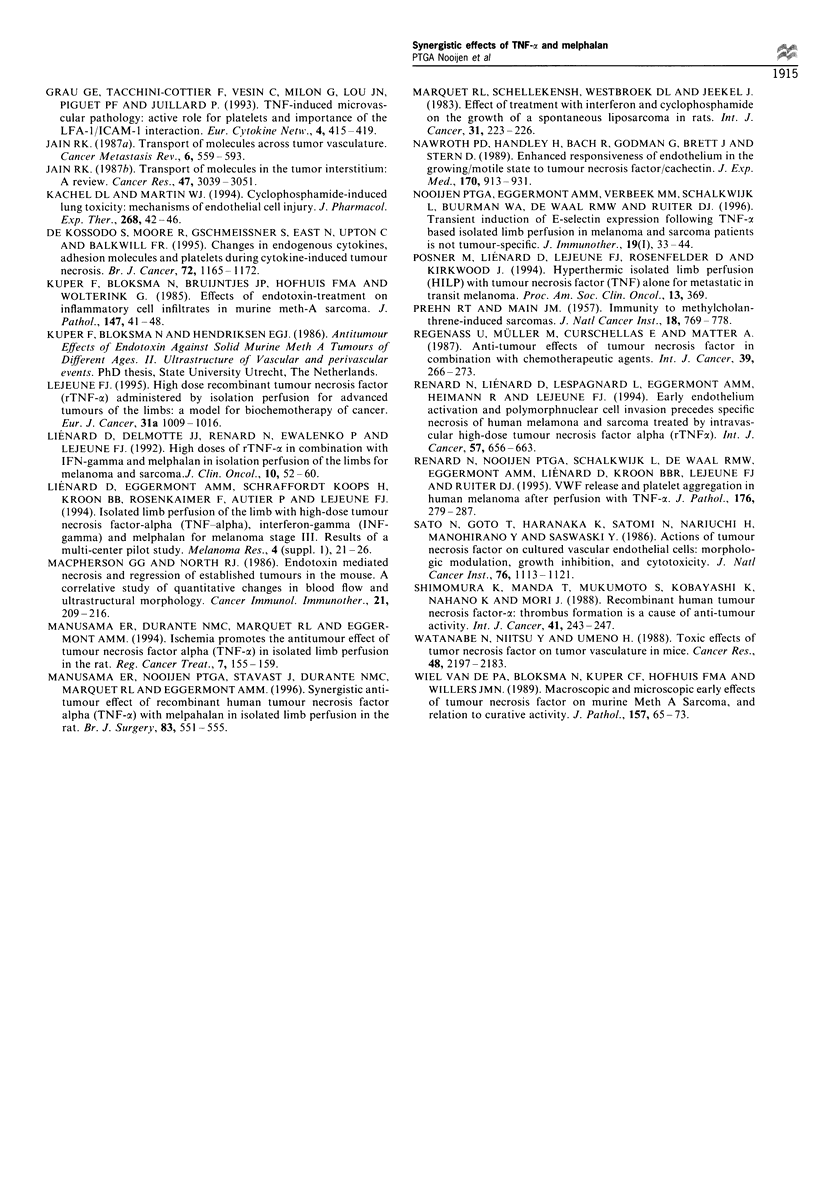


## References

[OCR_00867] Alexander R. B., Nelson W. G., Coffey D. S. (1987). Synergistic enhancement by tumor necrosis factor of in vitro cytotoxicity from chemotherapeutic drugs targeted at DNA topoisomerase II.. Cancer Res.

[OCR_00871] Asher A., Mulé J. J., Reichert C. M., Shiloni E., Rosenberg S. A. (1987). Studies on the anti-tumor efficacy of systemically administered recombinant tumor necrosis factor against several murine tumors in vivo.. J Immunol.

[OCR_00879] Bagchus W. M., Jeunink M. F., Rozing J., Elema J. D. (1989). A monoclonal antibody against rat platelets. I. Tissue distribution in vitro and in vivo.. Clin Exp Immunol.

[OCR_00884] Benckhuysen C., Van Dijk W. J., Van't Hoff S. C. (1982). High-flow isolation perfusion of the rat hind limb in vivo.. J Surg Oncol.

[OCR_00887] Bertomeu M. C., Gallo S., Lauri D., Levine M. N., Orr F. W., Buchanan M. R. (1990). Chemotherapy enhances endothelial cell reactivity to platelets.. Clin Exp Metastasis.

[OCR_00999] Gerlach H., Lieberman H., Bach R., Godman G., Brett J., Stern D. (1989). Enhanced responsiveness of endothelium in the growing/motile state to tumor necrosis factor/cachectin.. J Exp Med.

[OCR_00903] Gerlach H., Lieberman H., Bach R., Godman G., Brett J., Stern D. (1989). Enhanced responsiveness of endothelium in the growing/motile state to tumor necrosis factor/cachectin.. J Exp Med.

[OCR_00913] Grau G. E., Tacchini-Cottier F., Vesin C., Milon G., Lou J. N., Piguet P. F., Juillard P. (1993). TNF-induced microvascular pathology: active role for platelets and importance of the LFA-1/ICAM-1 interaction.. Eur Cytokine Netw.

[OCR_00921] Jain R. K. (1987). Transport of molecules across tumor vasculature.. Cancer Metastasis Rev.

[OCR_00925] Jain R. K. (1987). Transport of molecules in the tumor interstitium: a review.. Cancer Res.

[OCR_00929] Kachel D. L., Martin W. J. (1994). Cyclophosphamide-induced lung toxicity: mechanism of endothelial cell injury.. J Pharmacol Exp Ther.

[OCR_00940] Kuper C. F., Bloksma N., Bruyntjes J. P., Hofhuis F. M., Wolterink G. (1985). Effects of endotoxin-treatment on inflammatory cell infiltrates in murine Meth A sarcoma.. J Pathol.

[OCR_00952] Lejeune F. J. (1995). High dose recombinant tumour necrosis factor (rTNF alpha) administered by isolation perfusion for advanced tumours of the limbs: a model for biochemotherapy of cancer.. Eur J Cancer.

[OCR_00956] Lienard D., Ewalenko P., Delmotte J. J., Renard N., Lejeune F. J. (1992). High-dose recombinant tumor necrosis factor alpha in combination with interferon gamma and melphalan in isolation perfusion of the limbs for melanoma and sarcoma.. J Clin Oncol.

[OCR_00965] Liénard D., Eggermont A. M., Schraffordt Koops H., Kroon B. B., Rosenkaimer F., Autier P., Lejeune F. J. (1994). Isolated perfusion of the limb with high-dose tumour necrosis factor-alpha (TNF-alpha), interferon-gamma (IFN-gamma) and melphalan for melanoma stage III. Results of a multi-centre pilot study.. Melanoma Res.

[OCR_00972] MacPherson G. G., North R. J. (1986). Endotoxin-mediated necrosis and regression of established tumours in the mouse. A correlative study of quantitative changes in blood flow and ultrastructural morphology.. Cancer Immunol Immunother.

[OCR_00979] Manusama E. R., Nooijen P. T., Stavast J., Durante N. M., Marquet R. L., Eggermont A. M. (1996). Synergistic antitumour effect of recombinant human tumour necrosis factor alpha with melphalan in isolated limb perfusion in the rat.. Br J Surg.

[OCR_00992] Marquet R. L., Schellekens H., Westbroek D. L., Jeekel J. (1983). Effect of treatment with interferon and cyclophosphamide on the growth of a spontaneous liposarcoma in rats.. Int J Cancer.

[OCR_01005] Nooijen P. T., Eggermont A. M., Verbeek M. M., Schalkwijk L., Buurman W. A., de Waal R. M., Ruiter D. J. (1996). Transient induction of E-selectin expression following TNF alpha-based isolated limb perfusion in melanoma and sarcoma patients is not tumor specific.. J Immunother Emphasis Tumor Immunol.

[OCR_01017] PREHN R. T., MAIN J. M. (1957). Immunity to methylcholanthrene-induced sarcomas.. J Natl Cancer Inst.

[OCR_01019] Regenass U., Müller M., Curschellas E., Matter A. (1987). Anti-tumor effects of tumor necrosis factor in combination with chemotherapeutic agents.. Int J Cancer.

[OCR_01028] Renard N., Liénard D., Lespagnard L., Eggermont A., Heimann R., Lejeune F. (1994). Early endothelium activation and polymorphonuclear cell invasion precede specific necrosis of human melanoma and sarcoma treated by intravascular high-dose tumour necrosis factor alpha (rTNF alpha).. Int J Cancer.

[OCR_01033] Renard N., Nooijen P. T., Schalkwijk L., De Waal R. M., Eggermont A. M., Liénard D., Kroon B. B., Lejeune F. J., Ruiter D. J. (1995). VWF release and platelet aggregation in human melanoma after perfusion with TNF alpha.. J Pathol.

[OCR_01042] Sato N., Goto T., Haranaka K., Satomi N., Nariuchi H., Mano-Hirano Y., Sawasaki Y. (1986). Actions of tumor necrosis factor on cultured vascular endothelial cells: morphologic modulation, growth inhibition, and cytotoxicity.. J Natl Cancer Inst.

[OCR_01049] Shimomura K., Manda T., Mukumoto S., Kobayashi K., Nakano K., Mori J. (1988). Recombinant human tumor necrosis factor-alpha: thrombus formation is a cause of anti-tumor activity.. Int J Cancer.

[OCR_01058] Van de Wiel P. A., Bloksma N., Kuper C. F., Hofhuis F. M., Willers J. M. (1989). Macroscopic and microscopic early effects of tumour necrosis factor on murine Meth A sarcoma, and relation to curative activity.. J Pathol.

[OCR_01053] Watanabe N., Niitsu Y., Umeno H., Kuriyama H., Neda H., Yamauchi N., Maeda M., Urushizaki I. (1988). Toxic effect of tumor necrosis factor on tumor vasculature in mice.. Cancer Res.

[OCR_00934] de Kossodo S., Moore R., Gschmeissner S., East N., Upton C., Balkwill F. R. (1995). Changes in endogenous cytokines, adhesion molecules and platelets during cytokine-induced tumour necrosis.. Br J Cancer.

